# Late chronotype predicts more depressive symptoms in bipolar disorder over a 5 year follow-up period

**DOI:** 10.1186/s40345-021-00233-5

**Published:** 2021-09-01

**Authors:** Parisa Vidafar, Anastasia K. Yocum, Peisong Han, Melvin G. McInnis, Helen J. Burgess

**Affiliations:** 1 Sleep and Circadian Research Laboratory, Department of Psychiatry, University of Michigan, Rachel Upjohn Building, 4250 Plymouth Rd, Ann Arbor, MI 48109 USA; 2 Depression Center, Department of Psychiatry, University of Michigan, Ann Arbor, MI 48109 USA; 3 Department of Biostatistics, School of Public Health, University of Michigan, Ann Arbor, MI 48109 USA

**Keywords:** Chronotype, Circadian, Mood

## Abstract

**Background:**

There is increasing evidence that bipolar disorder is influenced by circadian timing, including the timing of sleep and waking activities. Previous studies in bipolar disorder have shown that people with later timed daily activities, also known as late chronotypes, are at higher risk for subsequent mood episodes over the following 12–18 months. However, these studies were limited to euthymic patients and smaller sample sizes. The aim of the current study was to further examine baseline chronotype as a potentially important predictor of mood-related outcomes in a larger sample of individuals with bipolar disorder and over the longest follow up period to date, of 5 years. Participants included 318 adults diagnosed with bipolar I and II (19–86 years) who were enrolled in the Prechter Longitudinal Study of Bipolar Disorder.

**Results:**

Participants with a late chronotype were found to be more likely to have mild to more severe depressive symptoms (PHQ-9 ≥ 5) as captured with PHQ-9 assessments every 2 months over the 5 year follow up period. This higher risk for depressive symptoms remained even after adjusting for age, sex and mood at baseline. Additionally, late chronotypes reported fewer hypomania/mania episodes during the 5 year follow up, as derived from clinical interviews every two years.

**Conclusions:**

These results highlight the potential clinical usefulness of a single self-report question, in identifying patients at risk for a more depressive mood course. The results also suggest that circadian phase advancing treatments, that can shift circadian timing earlier, should be explored as a means to reduce depressive symptoms in late chronotypes with bipolar disorder.

## Background

Bipolar disorder is characterized by pathological mood swings ranging from mania (e.g., high levels of energy, euphoria and minimal sleep need) to depression (e.g., low levels of energy, despair and increased sleep need or disrupted sleep) that are typically periodic but may become chronic (American Psychiatric Association DSM Task Force 2013). Bipolar I and bipolar II affects approximately 2% of the adult population in the United States (Merikangas et al. 2007; Miller et al. 2014), and suicide rates among individuals with bipolar disorder are 15 times higher than the general population (American Psychiatric Association DSM Task Force 2013). The etiology of bipolar disorder is unknown, but likely to be multifaceted (Harrison et al. 2018) and growing evidence suggests that bipolar disorder and the circadian system are intricately linked (Melo et al. 2017).

The gold standard measure of circadian timing in humans is the onset of melatonin secretion, when measured in dim light conditions (dim light melatonin onset, DLMO) (Klerman et al. 2002; Lewy et al. 1999). The DLMO typically occurs 2–3 h before an individual’s habitual sleep onset (Burgess and Fogg 2008), but melatonin must be measured in dim light as otherwise its secretion is suppressed by light (Lewy et al. 1980). The DLMO can be obtained non-invasively from half-hourly or hourly saliva samples, collected in the 6 h or so before habitual sleep onset (Burgess and Fogg 2008). However, the procedures to assess the DLMO requires staff support, considerable participant effort, and the melatonin assay is relatively expensive (Kantermann et al. 2015).

An alternative to measuring the DLMO is to simply ask people about the timing of their behaviors such as sleep and waking activities (their “chronotype”) using questionnaires such as the Morningness-Eveningness Questionnaire (MEQ) (Horne and Ostberg 1976) or the Munich ChronoType Questionnaire (MCTQ) (Roenneberg et al. 2003). Previous work has shown that chronotype derived from these questionnaires correlates with the DLMO (r = 0.70), and thus self-reported chronotype can serve as a proxy measure of circadian timing in humans (Kantermann et al. 2015). As with the DLMO, chronotype usually changes as a function of age. Children demonstrate morningness, or earlier circadian timing, but with the onset of puberty there is more eveningness, or later circadian timing. Eveningness peaks in the early twenties, before a steady return to morningness with increasing age (Fischer et al. 2017). There are also important sex differences in chronotype, with women typically showing earlier circadian timing and more morningness than men, although this difference diminishes after menopause (Fischer et al. 2017; Duffy et al. 2011).

Eveningness, or late chronotype, is well-recognized to be associated with more depressive symptoms in individuals with major depressive disorder and healthy controls (Kitamura et al. 2010; Levandovski et al. 2011; Merikanto et al. 2013; Selvi et al. 2010). People with bipolar disorder have more eveningness than healthy controls (Ahn et al. 2008; Wood et al. 2009; Baek et al. 2016; Mansour et al. 2005; Romo-Nava et al. 2020), even when euthymic (Giglio et al. 2010). Further, more eveningness in people with bipolar disorder was found to be associated with worse depressive symptoms (Wood et al. 2009) and a history of rapid mood swings (Mansour et al. 2005; Romo-Nava et al. 2020). Only two studies have assessed the influence of chronotype on subsequent mood course in bipolar disorder. One study found that in a sample of 104 euthymic individuals with bipolar disorder, those that met criteria for delayed sleep phase disorder (extreme eveningness), were more likely to have a mood episode in the following year (Takaesu et al. 2017). The other study examined baseline chronotype in 80 euthymic individuals with bipolar disorder and measured their subsequent anxiety, behavioral function, and number of mood episodes every month across an 18-month period. Eveningness at baseline significantly predicted poorer behavioral function, increased anxiety levels and a greater number of mood episodes (Melo et al. 2020). These results suggest that assessing chronotype in individuals with bipolar disorder may help identify patients at higher risk for adverse outcomes. As the authors of these previous studies acknowledged, 12–18-months were relatively short periods to assess mood course and the sample sizes were relatively small (Takaesu et al. 2017; Melo et al. 2020). Therefore, the aim of the current study was to further examine baseline chronotype as a potentially important predictor of mood-related outcomes in a larger sample of individuals with bipolar disorder over a longer follow up period. In addition, age and sex were included as important covariates known to impact both chronotype and mood.

## Methods

### Participants

Participants included 318 adults diagnosed with bipolar I and II, aged 19–86 years (see Table 1 for demographics and baseline psychosocial characteristics). All participants were enrolled in the Prechter Longitudinal Study of Bipolar Disorder (“Prechter study”), an observational, open cohort study established in 2005 at the University of Michigan, and had been followed for at least 5 years. The follow up analysis was restricted to 5 years to control for the varying duration of the follow up period across participants. The Prechter study gathers phenotypic and biological data with the intention of better understanding the underlying mechanisms of bipolar disorder (McInnis et al. 2018). Participants are continuously recruited into the study by way of online and print advertisements in inpatient and outpatient psychiatric clinics within the University of Michigan Health System, as well as mental health centers and outreach events within the broader community. At enrolment, bipolar I participants were required to have had a history of treatment for a manic episode, and for bipolar II a history of recurrent depression in addition to hypomania. They were assessed via the Diagnostic Interview for Genetic Studies (Nurnberger et al. 1994) and a best estimate process by at least two doctoral level clinicians was used to confirm diagnosis of bipolar I or II using the Diagnostic and Statistical Manual of Mental Disorders (DSM-IV) criteria. All participants were free from neurological disease and active substance dependence at enrollment. Participants provided written informed consent and all study procedures were approved by the University of Michigan Institutional Review Board.

**Table 1:** Participant demographics and psychosocial characteristics at baseline

Baseline characteristics (n = 318)	
Age (SD) years	43.7 (13.3)
Sex	
Females (number, %)	223 (70%)
Males (number, %)	95 (30%)
Chronotype, n = 318	
Early chronotype (number, %)	135 (42%)
Normal chronotype (number %) Late chronotype (number, %)	36 (11%) 147 (46%)
PHQ-9 (mean, SD) n = 297	8.0 (6.4)
PHQ-9 ≥ 5 (number, %)	190 (64%)
GAD-7 (mean, SD) n = 221	5.8 (4.4)
GAD-7 ≥ 10 (number, %)	48 (22%)
HAM-D (mean, SD) n = 265	2.4 (0.7)
HAM-D ≥ 10 (number, %)	100 (38%)
YMRS (mean, SD) n = 290	3.2 (4.5)
YMRS ≥ 12 (number, %)	15 (5%)

The mean and SD of each measure at baseline is listed

The sample size and percentage of the sample meeting the defined cut-off score at baseline is also described

### Measures

#### Self-report questionnaires

Chronotype at enrolment was collected using the original version of the Munich Chronotype Questionnaire (Roenneberg et al. 2003). In this questionnaire, one item asks people to rate their chronotype according to a scale (0 = extreme early type, 1 = moderate early type, 2 = slight early type, 3 = normal type, 4 = slight late type, 5 = moderate late type and 6 = extreme late type) which results in seven possible chronotypes. As this item was reported to be in excellent agreement with the total questionnaire score (Roenneberg et al. 2003), which is not easily derived (Kantermann et al. 2015), and the single item could be easily used in clinical practice, the utility of this single item was explored here. The 7 categories were combined into three groups for analysis: (1) early (includes extreme early, moderate early and slight early types, (2) normal, and (3) late (includes slight late, moderate late, and extreme late types).

Depressive symptoms were assessed with the Patient Health Questionnaire (PHQ-9), which is designed to determine the presence and severity of depressive symptoms over the previous two-week period (Kroenke et al. 2001). The PHQ-9 was initially collected at enrollment (“baseline”) and thereafter administered every two months. A score ≥ 5 was used to determine the presence of mild or more severe depressive symptoms.

The Generalized Anxiety Disorder Scale (GAD-7) was administered at enrollment and then every two months to assess for the severity of anxiety symptoms over the past two weeks (Spitzer et al. 2006). A score ≥ 10 was used to determine the presence of moderate or higher levels of anxiety.

#### Clinician administered interviews

The Hamilton Depression Rating Scale (HAM-D) was used to assess depressive symptoms at enrollment and thereafter every two years. The HAM-D is typically considered the gold standard assessment for measuring the presence and severity of depressive symptoms over the past week (Hamilton 1960; Rohan et al. 2016). A score ≥ 10 was used to determine the presence of depressive symptoms (Hamilton 1960).

The Young Mania Rating Scale (YMRS) was administered at enrollment and thereafter annually to assess manic symptoms. During the YMRS interview, individuals were asked about how they have been feeling over the past week. A score ≥ 12 was used to determine the presence of some manic symptoms (Young et al. 1978).

Every two years after enrollment, participants were interviewed with the Longitudinal Interval Follow-up Evaluation (LIFE), which measures the course of psychiatric disorders (Keller et al. 1987). From the LIFE interview several additional measures were derived: the number of hypomania/mania episodes, the number of depressive episodes, the number of hospitalizations and the number of suicide attempts, that had occurred since the last LIFE assessment.

#### Data analysis

To characterize mood-related outcomes following the baseline assessment, the number of assessments above a set score were divided by the total number of assessments of that same measure, for each participant. This generated a proportion metric for each measure and for each participant, whereby a higher proportion indicated a worse mood-related outcome. This approach helped account for the different frequencies with which the measures were assessed (from every two months to two years). So for example, for depressive symptoms, the number of assessments where PHQ-9 was ≥ 5 was divided by the total number of PHQ-9 assessments for each participant. Similarly, the number of assessments with a HAM-D score ≥ 10 was divided by the total number of HAM-D assessments. For anxiety symptoms, the number of assessments with GAD-7 ≥ 10 were divided by the total number of GAD-7 assessments. For manic symptoms, the number of assessments where YMRS ≥ 12 was divided by the total number of YMRS assessments. The LIFE interview outcomes were coded as the number of hypomania/mania episodes and depressive episodes during the follow up period, and whether at least one psychiatric hospitalization or suicide attempt had occurred during the follow up (to account for the low rate of psychiatric hospitalizations and suicide attempts during the follow up).

The influence of chronotype on subsequent mood-related outcomes estimated with a proportion were analyzed with a multivariate Poisson regression using “normal chronotype” as a reference group. The Poisson regression model explicitly takes the total number of assessments for each measure into account. The model included adjustment for age and sex (females used as a reference group) which are important influences on chronotype and mood. The model also adjusted for the baseline score, to determine if the assessment of chronotype provided any additional predictive utility for mood-related outcomes, above and beyond the baseline score of that specific measurement tool. For the LIFE interview measures of number of mood episodes, and whether a psychiatric hospitalization or suicide attempt had occurred at all during the follow up period, the data was analyzed using logistic regression. Baseline YMRS score was included in the models assessing hypomania/mania, suicide attempts and psychiatric hospitalizations, and baseline PHQ-9 score was included in the models assessing depressive episodes, suicide attempts and psychiatric hospitalizations. All of the data was analyzed using the R software (https://cran.r-project.org/).

## Results

**Fig. 1:**
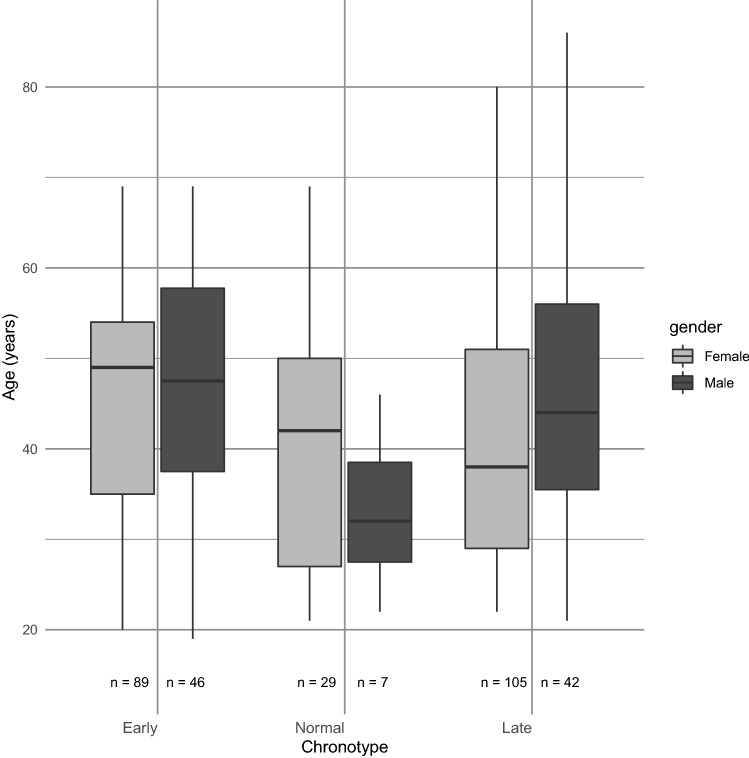
Boxplots of age is shown for each sex within each of the three chronotypes. The maximum and minimum age is shown by the top and bottom of the vertical lines, the interquartile range is represented by the box, and median by the horizontal line. Compared to early chronotypes, late chronotypes were more likely to be younger and male

The sample of bipolar I and II participants were mostly female, and on average middle aged (Table 1). Only about 1 in 10 participants identified as having a normal chronotype, with most reporting either a late or early chronotype. The distribution of chronotype according to age and sex in the sample indicated that at least compared to early chronotypes, the late chronotypes were younger, with almost half of males and females being a late chronotype (Fig. 1).

At baseline, almost two-thirds of the sample reported at least some mild depressive symptoms on the PHQ-9. Fewer participants were identified with depressive symptoms using the HAM-D, which may have reflected the higher cut-off score of 10 or more. About a quarter of the sample reported moderate anxiety symptoms on the GAD-7. Only a small minority of participants had manic symptoms at baseline, as reflected on the YMRS. Some participants did not complete all scheduled assessments, and the final sample size for each measure is shown in Table 1. Missing data ranged from 6.6% for the PHQ-9 to 30.5% for the GAD-7, mostly due to the GAD-7 not being included in the Prechter study until March 2012. The average number of follow up assessments per participant were 22.6 for the PHQ-9, 9.9 for the GAD-7, 2.4 for the HAM-D and 3.6 for the YMRS. On average each participant completed 2.4 LIFE interviews.

**Fig. 2:**
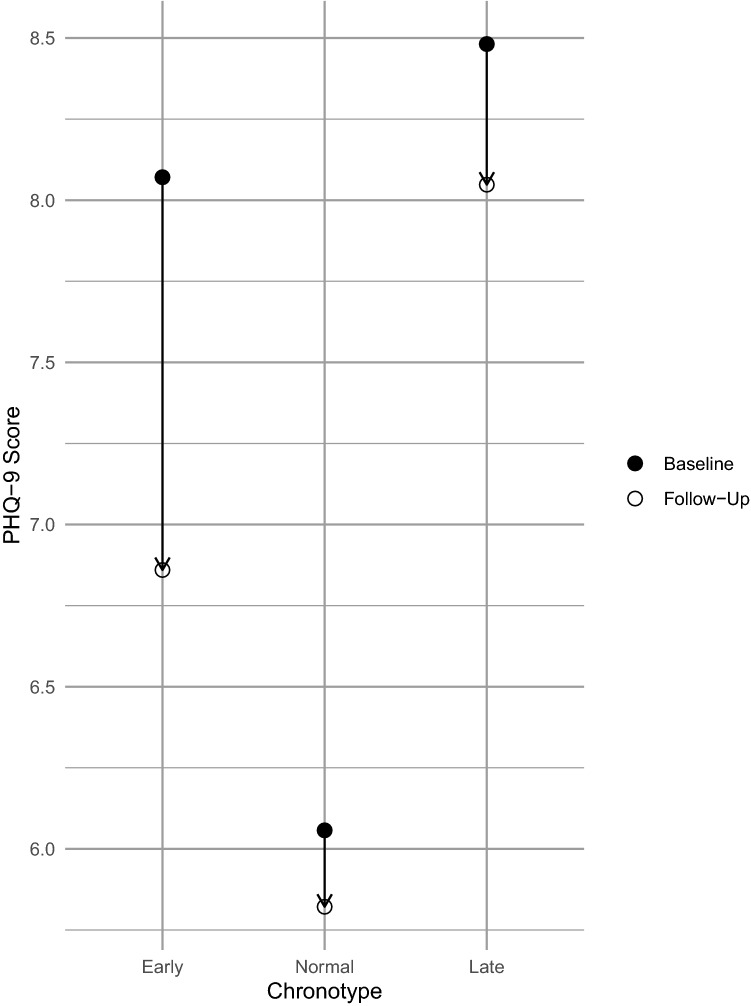
The mean baseline PHQ-9 score and mean follow up PHQ-9 score of early, normal, and late chronotypes

The regression results indicated that younger adults with bipolar disorder I and II were more likely to have depressive and anxiety symptoms on the PHQ-9 and GAD-7 during the follow up period, although the magnitude of this effect was very small and likely not clinically meaningful (Table 2). Similarly, those participants with higher scores on the GAD-7, HAM-D and YMRS at baseline were slightly more likely to have a higher proportion of assessments with GAD-7 ≥ 10, HAM-D ≥ 10 and YMRS ≥ 12 during the follow up period. Late chronotype predicted a higher proportion of assessments of PHQ-9 ≥ 5 during the follow up period, even after adjusting for age, sex and baseline score. On average in all chronotypes, there was a decrease from the baseline PHQ-9 to the average PHQ-9 during follow up, reflecting a reduction in depressive symptoms while participating in the Prechter study (Fig. 2). Notably, as compared to the early and normal chronotypes, the late chronotypes started with the highest average PHQ-9 at baseline and decreased on average by less than 0.5 during the follow up period to reach close to the average baseline PHQ-9 score of the early chronotypes.

**Table 2:** Results of a model using age, sex, baseline score and chronotype to predict the rate of mood assessments above a predefined score during the follow up period

	Rate ratio [95% CI]				
	Age (years)	Sex (male)	Baseline score	Early chronotype	Late chronotype
PHQ-9 ≥ 5	**0.995** **[0.992–0.997]**	1.069 [0.980- 1.166]	0.996 [0.990–1.00]	1.115 [0.977—1.272]	**1.290** **[1.135–1.466]**
GAD-7 ≥ 10	**0.995** **[0.992–0.998]**	0.945 [0.854–1.045]	**1.034** **[1.024–1.043]**	1.003 [0.871–1.155]	1.034 [0.903–1.185]
HAM-D ≥ 10	1.002 [0.993–1.012]	1.162 [0.905–1.492]	**1.084** **[1.071–1.098]**	0.826 [0.564–1.210]	0.794 [0.547–1.151]
YMRS ≥ 12	1.001 [0.985–1.018]	0.822 [0.525–1.286]	**1.164** **[1.133–1.195]**	0.973 [0.485–1.951]	0.968 [0.486–1.929]

Females and normal chronotype were used as reference groups

Bold RRs are significant at p < 0.01

**Table 3:** Results of a model using age, sex, baseline YMRS score and chronotype to predict the number of hypomania/mania episodes during the follow up period as derived from the LIFE interview

	Rate ratio [95% CI]				
	Age (years)	Sex (male)	YMRS	Early chronotype	Late chronotype
Hypomania/mania	0.999 [0.995–1.002]	**1.142** **[1.034–1.260]**	**1.050** **[1.041–1.060]**	0.921 [0.797–1.065]	**0.747** **[0.646–0.863]**

Females and normal chronotype were used as reference groups

Bold RRs are significant at p < 0.01

**Table 4:** Results of a model using age, sex, baseline PHQ-9 score and chronotype to predict the number of depressive episodes during the follow up period as derived from the LIFE interview

	Rate ratio [95% CI]				
	Age (years)	Sex (male)	PHQ-9	Early chronotype	Late chronotype
Depression	0.997 [0.992–1.001]	0.961 [0.848–1.090]	**1.051** **[1.043–1.059]**	0.984 [0.825–1.173]	0.946 [0.796–1.124]

Females and normal chronotype were used as reference groups

Bold RRs are significant at p < 0.01

**Table 5:** Results of a model using age, sex, chronotype, baseline PHQ-9 and YMRS scores to predict occurrence of a suicide attempt or psychiatric hospitalization during the follow up period as derived from the LIFE interview

	Odds ratio [95% ci]					
	Age (years)	Sex (male)	PHQ-9	YMRS	Early chronotype	Late chronotype
Suicide attempts	1.012 [0.976–1.048]	0.513 [0.145–1.820]	1.057 [0.997–1.120]	0.954 [0.836–1.089]	0.610 [0.181–2.054]	0.539 [0.165–1.767]
Psychiatric hospitalizations	0.988 [0.972–1.004]	**0.321** **[0.166–0.619]**	**1.067** **[1.040–1.095]**	1.020 [0.971–1.066]	1.052 [0.568–1.948]	0.877 [0.482–1.594]

Females and normal chronotype were used as reference groups

Bold ORs are significant at p < 0.01

In terms of the LIFE interview variables, hypomania/mania episodes were less frequent in late chronotypes, and hypomania/mania episodes were more frequent in males and in those with a higher baseline YMRS score (Table 3). Depressive episodes were slightly more likely to occur in those with a higher baseline PHQ-9 score. No effects of age, sex, baseline scores or early or late chronotype were observed on the occurrence of a suicide attempt. However, males and those with a lower PHQ-9 score at baseline were less likely to have a psychiatric hospitalization during the follow up period (Table 4).

## Discussion

The results of this study indicate that people with bipolar I and II who self-identify as late chronotypes, have a higher frequency of depressive symptoms over a 5 year follow up. Thus bipolar patients with late chronotype are at increased risk for a more depressive mood course over time, even after adjusting for the effects of age, sex and mood at baseline. This result was observed with the bimonthly administration of the PHQ-9, but not with the HAM-D or LIFE interviews. The HAM-D assessed depressive symptoms over the past week, but was only assessed every 2 years and therefore could have more easily missed detecting depressive symptoms. The LIFE interview would have captured depressive episodes, but does rely on participant recall of the previous 2 years, and detects depressive episodes, which are likely more severe than the mild depressive symptoms detected with a PHQ-9 threshold of 5. Notably the average PHQ-9 score at baseline and follow up in the late chronotype group remained under 10 (Fig. 2), further highlighting that the depressive symptoms were on average, mild. Interestingly, late chronotype was also associated with a reduced likelihood of hypomania/mania episodes on the LIFE interview, an effect that was also seen with the YMRS, although this was not statistically significant. This result raises the interesting possibility that late chronotype could be protective against manic relapse and symptoms, which should be explored in further research (Table 5).

This study of the impact of chronotype on subsequent mood course in people with bipolar disorder is the largest study to date, in a sample three times that of previous studies and with a follow up period more than three times as long (Takaesu et al. 2017; Melo et al. 2020). In both previous studies, late chronotype predicted more mood episodes in the following 12–18 months, but hypomania/mania episodes or symptoms were not distinguished from depressive episodes or symptoms due to limited statistical power. Our results suggest that late chronotype predominantly increases the risk for a depressive mood course rather than a hypomania/mania mood course. One previous study also reported that late chronotypes had more anxiety symptoms during follow up (Melo et al. 2020), but this effect was not detected in the current study possibly due to the smaller number of GAD-7 assessments. Both previous studies only included individuals who were euthymic at baseline, whereas in the current study all participants were included and baseline mood was adjusted for in the analyses. Thus the current results are relevant to any patient with bipolar disorder regardless of current mood state, and not only to euthymic patients.

The association between late chronotype (reflecting later or delayed circadian timing) and a greater likelihood of depressive symptoms is not unique to bipolar disorder, having been observed in individuals with major depressive disorder and even in healthy controls (Kitamura et al. 2010; Levandovski et al. 2011; Merikanto et al. 2013; Selvi et al. 2010). Indeed, there is preliminary evidence from a double-blinded randomized study in healthy controls, that even small circadian phase delays of only 30 min, can reduce people’s overall sense of well-being (Yang et al. 2001). Others have reported that people with bipolar disorder have more eveningness, or are more likely to be a late chronotype, than healthy controls (Ahn et al. 2008; Wood et al. 2009; Baek et al. 2016; Mansour et al. 2005), even when they are euthymic (Giglio et al. 2010). Furthermore, when chronotype was assessed in people with bipolar disorder at two to three time points over two to four years, evening or late chronotype was found to be a stable characteristic, irrespective of mood state (Wood et al. 2009; Seleem et al. 2015). Together, these findings suggest the possibility that eveningness or late chronotype may be a trait marker of bipolar disorder (Seleem et al. 2015).

The finding that people with bipolar disorder who self-identified as being a late chronotype, had a more depressive mood course over the following 5 years, even after adjusting for sex, age and baseline score, raises the possibility that treatments that phase advance or shift circadian timing earlier, may help reduce depressive symptoms in people with bipolar disorder. Indeed, others have suggested circadian timing as a target for improving the mood course in bipolar disorder (Takaesu et al. 2017). Morning light treatment is well-recognized to phase advance circadian rhythms, and has antidepressant properties (Al-Karawi and Jubair 2016). Morning bright light treatment has been found to be antidepressant in people with bipolar disorder, but concerns over inducing mania have led to recommendations to administer light treatment in the middle of the day rather than the morning (Sit et al. 2018), the timing of which does not typically lead to large phase advances (Khalsa et al. 2003). Interestingly, low doses of melatonin (e.g. 0.5 mg) in the few hours before habitual bedtime are well-recognized to phase advance the circadian clock without the sleepiness side effect often associated with melatonin (Burgess et al. 2010). While higher doses of melatonin taken at bedtime have been tested in people with bipolar disorder as a sleep aid (Baandrup et al. 2011) and to improve cardiometabolic health (Mostafavi et al. 2017), it remains to be determined if melatonin, when used to phase advance circadian timing, can improve mood course in people with bipolar disorder. While the five years of follow up in this study is meaningfully longer than the previous studies (Takaesu et al. 2017; Melo et al. 2020), the granularity and frequency of the data collection nonetheless remains modest. The gathering of self-reported mood symptoms every two months and an in-person clinical assessment every two years may still have not accurately captured the nuanced daily to weekly variability in mood in people with bipolar disorder. Additionally, the restriction of the data analysis to those participants with at least 5 years of follow up means that more recently enrolled participants in the Prechter study were not included in this study. Further, chronotype was self-reported and subject to bias at the individual level, with no objective markers of sleep or circadian timing to verify the self-report. For analytical reasons the chronotype categories were collapsed from seven to three groups, which may have obscured any differences due to the intensity of chronotype, such as between the extreme, moderate and slight late chronotypes. Lastly, we note only 11% of the participants identified as a ‘normal chronotype’, with the remainder of the sample reporting being some degree of an early type (42%) or late type (46%). This is not dissimilar to results from the largest cross-sectional study to date of chronotype in bipolar disorder (n = 773), where only 16% identified as being neither a morning or evening type, with the rest reporting as a morning type (35%) or evening type (49%) (Romo-Nava et al. 2020).

## Conclusions

This study replicates the findings that people with bipolar disorder who identify as late chronotypes have a worse mood course over the following years. Specifically, this study found that late chronotypes had a higher rate of mild or more severe depressive symptoms as assessed bimonthly with the PHQ-9 over 5 years. Conversely, late chronotypes were less likely to experience a hypomania/mania episode over 5 years. These results highlight the importance of frequent assessments with sensitive instruments over prolonged periods to better capture mood course in bipolar disorder over time. Future research could examine if the influence of late chronotype on disease course also extends to other significant life outcomes including productivity and employment.

## Data Availability

The datasets generated and/or analyzed during the current study are not publicly available due to privacy restrictions, but are available from the Prechter study on reasonable request.
